# Prefrontal TRPM8 Receptor Modulates Epileptic Seizures via PKA/CREB Signaling Pathway in Mice

**DOI:** 10.1002/cns.70709

**Published:** 2025-12-31

**Authors:** Jia‐Zhan Huang, Gui‐Feng Lu, Yi‐Han Jiang, Yao Guo, Ze‐Yu Lin, Zhi Zhang, Fei Geng

**Affiliations:** ^1^ Department of Stomatology Shantou University Medical College Shantou China; ^2^ Department of Physiology Shantou University Medical College Shantou China; ^3^ Department of Molecular Medicine Shantou University Medical College Shantou China

**Keywords:** CREB, PKA, seizures, TRPM8 receptor

## Abstract

**Aim:**

Epilepsy is a common neurological disorder accompanied by mental and cognitive impairment, which affects approximately 50 million people worldwide. Recent studies revealed that transient receptor potential melastatin 8 (TRPM8) receptors exerted a significant effect in PTZ‐induced acute seizure model. However, the exact function and mechanism of prefrontal TRPM8 receptor in seizures remain unclear. This study aimed to investigate the upstream and downstream signaling pathways of TRPM8 receptors and how they jointly regulate the occurrence and development of seizures.

**Methods:**

Pentylenetetrazol (PTZ) was used to establish an acute mouse seizure model, and the seizure behavior of TRPM8 channel block mice and normal mice was observed and analyzed. Specific blocking of TRPM8 channels in specific brain regions was performed by stereotactic injection into the brain. The expression of TRPM8 downstream signaling molecules in the prefrontal cortex (PFC) and the apoptosis of neuronal cells were analyzed after PTZ‐induced acute seizures.

**Results:**

TRPM8 receptors were upregulated in the PFC of mice with seizures. Inhibition or knockdown of TRPM8 in the PFC can effectively prolong the latency and reduce the level of seizures in mouse models induced by PTZ. Meanwhile, prefrontal TRPM8, Phosphorylated PKA (p‐PKA) and Phosphorylated CREB (p‐CREB) levels were upregulated during seizures. In the PTZ‐induced acute seizure cell model, the expression of TRPM8, p‐CREB, and p‐PKA was also increased, but this effect was reversed by the TRPM8 inhibitor AMTB. PKA agonists significantly offset the effects of TRPM8 inhibitors in prolonging latency and reducing seizure levels. Finally, TUNEL staining showed that the apoptosis rate of prefrontal neurons in seizure mice decreased after TRPM8 inhibition and knockdown, while PKA activation could counteract the AMTB‐induced decrease in neuronal apoptosis.

**Conclusion:**

Prefrontal TRPM8 receptor plays a vital role in PTZ‐induced acute seizures through the PKA/CREB pathway, which provides a potential target for the treatment of seizures.

## Graphical abstract

Jia‐zhan Huang et al. describe a function of prefrontal TRPM8 in the regulation of epileptic seizures. TRPM8 receptors was upregulated in the prefrontal cortex (PFC) of epileptic mice. Inhibition or knockdown of TRPM8 in PFC can effectively prolong the latency and reduce the level of seizures in mouse models induced by pentatetrazole. At the same time, a novel molecular mechanism has been elucidated that TRPM8 regulates seizures via the PKA/CREB signaling axis.

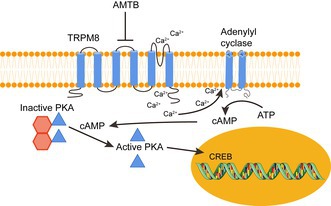

## 1 Introduction

Epilepsy is a ubiquitous neurological disorder from which approximately 50 million people worldwide suffer [1]. It affects a wide range of people, with the highest incidence among newborns and the elderly [2]. There are differences in the common causes of epilepsy in all ages. The main causes of epilepsy in children are congenital defects and genetics, and in adults they are brain injury, infection, and tumor, and in the elderly it is stroke, neurodegenerative diseases, or cardiovascular and cerebrovascular diseases [3]. Epilepsy causes more than just mental and cognitive impairment and is often accompanied by co‐morbidities. At the same time, epileptics are exposed to a certain risk of lifelong affliction [4]. Studies have shown that the mortality rate for people with epilepsy is eight times higher than that of the general population [5]. Structural changes, genetics, metabolic abnormalities, infections, and immune abnormalities can cause epilepsy [6]. Blockade or activation of both synaptic and voltage‐gated inhibitory transmission can acutely induce seizures, essentially causing an imbalance between excitation and inhibition [7]. Epilepsy not only causes health problems, but also reduces the quality of life and imposes great financial pressure on patients and their families. At present, the treatment of epilepsy mainly includes drug therapy and surgical treatment, among which antiepileptic drug therapy is the first‐line treatment of epilepsy. However, current drug treatments are associated with side effects, adverse reactions, drug resistance, and other problems [8]. Although studies have been reported on the causes and mechanisms of epilepsy, further exploration is indispensable to develop treatments and targeted therapies [9, 10]. Ion channels are the structural basis of the excitability regulation of excitable tissues in vivo, and are closely related to the pathogenesis of seizures. Previous studies have revealed that epileptic seizure is an ion channel disease, which may be caused by mutations in the genes encoding ion channel proteins, resulting in changes in the function of ion channels, causing changes in the excitability or inhibition of nerve activity, and eventually leading to seizures [11].

Transient receptor potential melastatin‐8 (TRPM8) channel, as a non‐selective Ca^2+^ channel, plays an important role as a cold thermoreceptor in the peripheral nervous system [12, 13]. TRPM8 channels are widely distributed and are expressed not only in various organs of the body and the peripheral nervous system but also in certain regions of the central nervous system, such as the thalamus, the hippocampus, the prefrontal lobe, and the amygdala [14, 15, 16]. A plethora of studies identified the significant role of TRPM8 in disorders of the genitourinary, respiratory, and immune systems, as well as persistent inflammatory activation in chronic inflammation [17]. The most fundamental function of TRPM8 is to allow Na^+^, K^+^, and Ca^2+^ to enter the cell, causing the cell to depolarize and generate an action potential that ultimately makes the organism feel cool or cold [18]. TRPM8 channels are activated at low temperature, menthol, voltage, and elevated osmolality [12]. TRPM8 activation was found to promote the transcription of c‐Fos, Egr‐1, and Elk‐1, genes that play important roles in neuronal excitability, synaptic plasticity, and long‐term memory consolidation and reconsolidation [19]. This previous study indicated a potential crucial role of TRPM8 in the regulation of brain function. TRPM8 has been found to lower core body temperature and play an anticonvulsant role in seizures [20, 21]. Nonetheless, there was a limited number of studies on the role of TRPM8 in seizures, and the mechanism of TRPM8 in seizures still needed to be further defined.

cAMP‐response element binding protein (CREB) is an essential element of multiple gene promoters and a key transcriptional regulator of the leucine zipper transcription factor family, which can recognize genes with AMP response element (CRE) sequences in the upstream regulatory region. It is required to activate gene transcription mediated by cAMP, an important signaling molecule in the nervous system [22]. The cAMP‐dependent protein kinase (PKA) was one of the imperative kinases of CREB, which activates CREB transcription by phosphorylating Ser^133^ of CREB [23]. In recent years, it has been found that CREB in the nervous system is involved in synaptic plasticity regulation, which is closely related to spatial memory and long‐term memory [24]. Meanwhile, it is involved in the development of neurodegenerative diseases such as Parkinson's disease and Alzheimer's disease [25, 26, 27]. Notably, CREB has been found to be constantly activated and increased in epileptogenesis [28]. CREB suppression has been shown to help reduce the duration of status epilepticus and the number of spontaneous seizures [29, 30]. However, the mechanism still remains unclear.

In the present study, we explored the role of prefrontal TRPM8 in epileptic seizures and its characteristics, and confirmed that blockade or knockdown of TRPM8 in the prefrontal lobe effectively alleviated the acute seizure progression. Given that TRPM8 and the PKA/CREB pathway have been found to be associated with diabetes and AML, respectively, the expression of TRPM8, CREB, and PKA in PFC was investigated in an acute seizure mouse model. We have shown that the levels of TRPM8, PKA, and CREB were increased during seizures. PKA agonists significantly offset the effects of TRPM8 inhibitors in prolonging latency and reducing seizure levels. Furthermore, TUNEL staining demonstrated that inhibition or knockdown of TRPM8 attenuated seizure‐induced neuronal apoptosis. Based on the above results, we proposed a new mechanism by which TRPM8 is involved in the regulation of the seizure process by regulating neuronal apoptosis through the PKA/CREB signaling pathway. Our findings provide potential targets for the treatment of epileptic seizures.

## 2 Materials and Methods

### 2.1 Animals

Mice in all experiments comprised male and female 8‐week‐old mice, including TRPM8‐WT (TRPM8 wild type) mice and TRPM8‐KO (TRPM8 knockdown) mice (20 g–30 g) from the Laboratory Animal Center of Shantou University Medical College. Mice were treated in a standard environment with a 12/12‐h light/dark cycle, a temperature of 22°C, and free access to water and food. Mice were randomly divided into different groups according to the experiential objective. The specific grouping situation is presented in the flow diagram (Figure S1).

### 2.2 Administration

For inhibition of TRPM8, TRPM8 inhibitor N‐(3‐aminopropyl)‐2‐{[(3‐methylphenyl)methyl]oxy}‐N‐(2‐thienylmethyl)benzamide (AMTB) hydrochloride salt (Tocris) was dissolved in 0.9% NaCl solution at a dose of 6 mg/kg, which was intraperitoneally injected into mice 30 min before modeling. The experiment involved randomly segregating the mice into two distinct groups: the AMTB group received AMTB treatment, the other group received injection of 0.9% NaCl solution.

### 2.3 Stereotactic Injection

The TRPM8‐WT mice were anesthetized using isoflurane and subsequently shaved to expose the surgical site. The mice were placed on the mice adaptor in the stereotactic apparatus and fixed. After disinfecting the area, a midline incision was made using surgical instruments to expose the skull and position bregma as the origin. For intracerebroventricular injections into the prefrontal lobe, the following coordinates were utilized: anteroposterior = 1.9 mm, mediolateral = 0.4 mm, and dorsoventral = 1.5 mm. The mice were implanted with cannula at the coordinates specified above, allowing for the free administration of substances during the formal experiment. A stylus was inserted into the guiding cannula to prevent clogging. Denture acrylic was cured to secure the guiding cannula in place. The mice were provided with a week's recovery period following surgery prior to the commencement of the formal experiment. Once all procedures were completed, the mice were placed on a heating pad after their incisions had been sutured. To facilitate the administration of drugs during free movement, the implanted cannula was connected to polyethylene tubing, which in turn was attached to a Hamilton syringe, enabling the injection of medication in freely moving subjects. The 6‐BNZ‐cAMP (N6‐benzoyladenosine‐3′,5′‐cyclic monophosphate sodium salt) solution at a concentration of 10 μg/μL was maintained at a constant infusion rate of 0.1 μL/min using an automatic pump, with a total volume of 800 nL. After the injection was finished, the needle was kept in place for 10 min for sufficient penetration. The mice were then placed back in their home cage for 30 min waiting for the modeling.

### 2.4 Settlement of PTZ‐Induced Model and Monitoring

Pentylenetetrazole (PTZ) was used to induce seizures in our study, and a specific antagonist, AMTB, was applied to inhibit TRPM8 in the PTZ‐induced acute seizure model. PTZ was dissolved in 0.9% saline at a concentration of 8 mg/mL and finally injected intraperitoneally into mice at 60 mg/kg. After administering AMTB and saline for 30 min, both groups were treated with PTZ intraperitoneally. To assess the behavioral changes, each mouse was observed individually in a transparent cage. The seizure behavior was monitored for 30 min and ranked according to the Racine scale.

1. Normal behavior;
2. Grade1 Ear and facial twitching;
3. Grade2 Myoclonic body jerks;
4. Grade3 Clonic forelimb convulsions;
5. Grade4 Generalized clonic convulsions and turning onto the side;
6. Grade5 Generalized clonic–tonic convulsions and loss of postural control;
7. Death

Recording the incubation period of each grade. To prevent errors, all the experimenters who observed and documented the mice behavior had undergone extensive observation and training to ensure they provided the most precise assessment of the rodents' actions. In this study, three experimenters rotated between different groups to guarantee the validity and dependability of the experimental outcomes.

### 2.5 Western Blotting

Brain tissue samples from the prefrontal lobe of the normal control group and the PTZ/saline group mice were taken respectively at days 1, 3, 5 after successfully modeling. Tissue samples were homogenized in RIPA, PMSF, PIC (RIPA: PMSF: PIC = 100:1:1) lysis. The samples were subsequently incubated on the ice for full dissociation. After standing for 30 min, supernatant was obtained by 12,000 r/15 min centrifugation. The protein concentration qualifications were performed by BCA protein assay kit. 60 μg of protein were electrophoretically separated on 10% SDS‐PAGE gels and transferred onto PVDF membranes (Millipore, IPVH00010). The PVDF membranes were subsequently blocked with 5% skimmed milk in PBST buffer for 1 h at room temperature (RT), followed by overnight incubation with primary antibodies at 4°C. Primary antibodies were used as follows: Phospho‐(Ser/Thr) PKA Substrate Antibody (Cell Signaling, 9621, 1:11000), Phospho‐CREB1‐S133 Rabbit pAb (ABclonal, AP0019, 1:1000), TRPM8 Polyclonal Antibody (Bioswamp, PAB37863, 1:1000), and β‐actin (Proteintech, 66009‐1‐Ig,1:20000). Finishing incubation with primary antibodies, the membranes were washed three times with PBST for 8 min each time. After incubation with goat anti‐rabbit, the membranes were incubated with horseradish peroxidase‐conjugated secondary antibodies (Abcam, ab6721, 1:10,000) for 1 h, at RT and washed 3 times with PBST. Immunoreactive bands were visualized using ECL reagents (Millipore, WBKLS0500). Image J software was applied to quantify the intensities of bands.

### 2.6 Cell Culture

The human neuroblastoma cell line SH‐SY5Y was maintained by the Department of Physiology, Shantou University Medical College. Cells were cultured under standard conditions: 10% FBS, at 37°C in a humidified atmosphere of 5% CO_2_. PTZ‐Induced TRPM8‐PKA/CREB Pathway Analysis: SH‐SY5Y cells were treated with 20 mM pentylenetetrazol (PTZ) for 48 h. The control group was given equivalent volume of saline. Cells were harvested for Western blot analysis of TRPM8, PKA, CREB. Pathway Rescue Validation: Cells were treated for 48 h in three groups, Control: saline; Seizure model: 20 mM PTZ; Therapeutic intervention: 20 mM PTZ + 2.0 μM AMTB (TRPM8 inhibitor). Lysates subjected to Western blot for p‐PKA and p‐CREB.

### 2.7 TUNEL Staining

One step TUNEL apoptosis assay kit (Beyotime) was utilized to detect the DNA fragmentation and apoptosis of neuron cells. Brain tissues were removed after 24 h of modeling via transcardiac perfusion‐fixation. The brains were subsequently fixed in a 4% paraformaldehyde solution overnight and then dehydrated using a sucrose solution. Serial sections, 10 μm thick, were prepared from the prefrontal lobe and re‐fixed in 4% paraformaldehyde to ensure optimal cell immobilization. Thereafter, they were washed with Phosphate Buffer (PBS) for 10 min twice. The sections washed with PBS were incubated in 0.5% Triton X‐100 PBS for 5 min and incubated in the prepared TUNEL detection solution for 60 min at 37°C in a humidified environment. Following incubation, the slices underwent three washes with PBS and were then prepared for microscopic examination. The slices were imaged using a fluorescence microscope (U‐HGLGPS, OLYMPUS, Japan) at a magnification of 40×. The green fluorescence apoptotic cells were identified as “TUNEL positive cells” and the numbers of TUNEL‐positive cells were calculated with ImageJ software (version 1.8.0).

### 2.8 Statistical Analysis

The data are presented as mean ± SEM. Statistical analysis was conducted using GraphPad Prism 9 software (GraphPad Software Inc., CA, USA). The Shapiro–Wilk test was employed to assess normality, while the Brown‐Forsythe test was utilized for examining variance homogeneity across multiple groups. Student's *t*‐test was applied for comparisons between two groups when the data adhered to a normal distribution. For data analysis among multiple groups, one‐way ANOVA was used. When the data did not conform to a normal distribution, the Mann–Whitney test was applied. In cases where a significant difference was detected using the ANOVA method, Fisher's Least Significant Difference (LSD) test was subsequently performed to calculate pairwise comparisons between means. In all statistical analyses, a *p*‐value < 0.05 was considered as the criterion for statistical significance.

## 3 Results

### 3.1 Inhibition of TRPM8 Alleviates the Progression of Acute Seizures

**FIGURE 1:**
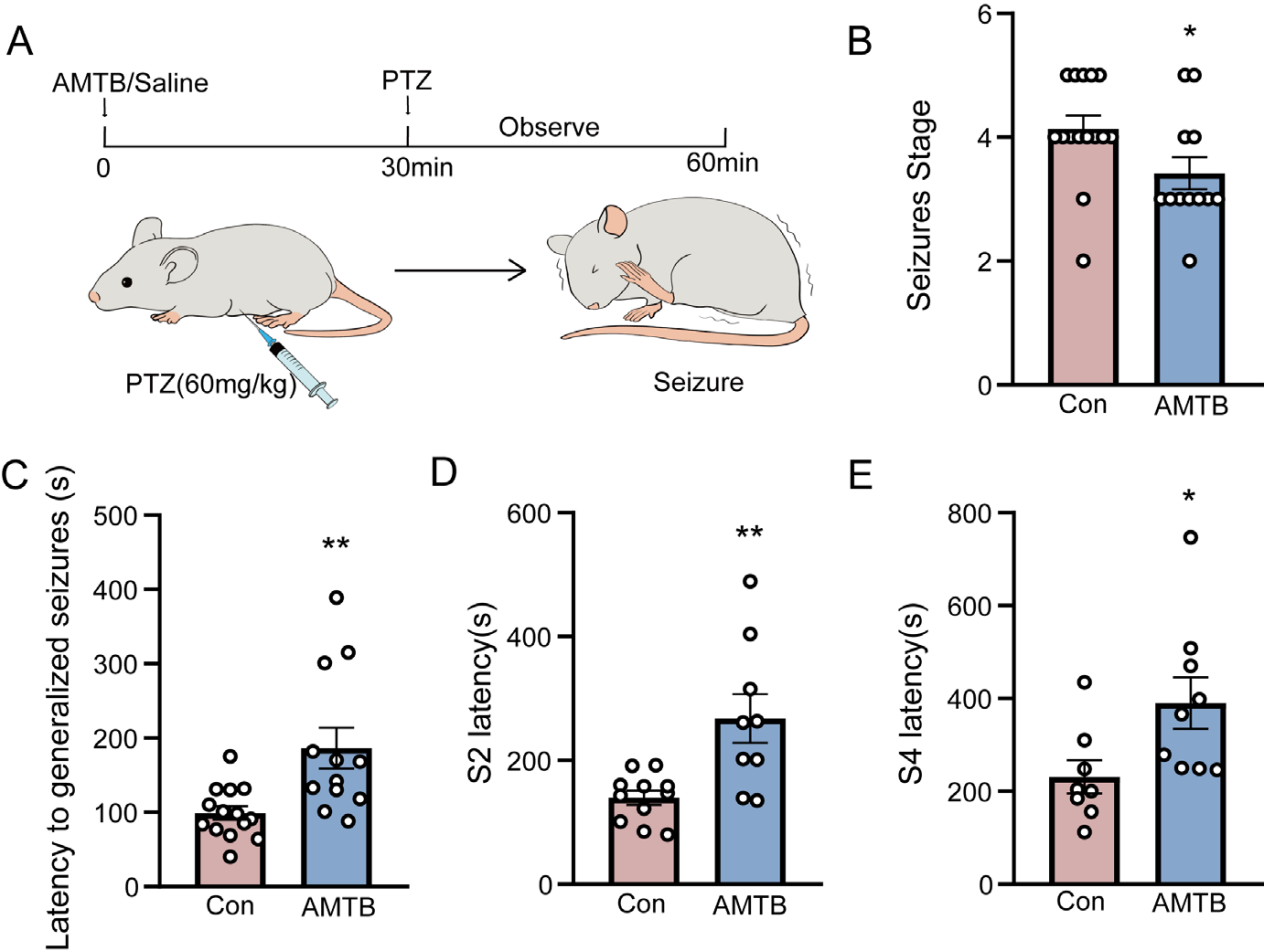
Inhibition of TRPM8 alleviates the acute seizure progression. (A) Schematic diagram of seizures induced by intraperitoneal injection of PTZ in mice. (B) Effect of intraperitoneal injection of the TRPM8 inhibitor AMTB on seizure stage in PTZ‐induced acute seizure mice (*n*: Con = 15, AMTB = 12, 4.13 ± 0.22 vs. 3.42 ± 0.26, *p* = 0.03). (C) Effect of intraperitoneal injection of the TRPM 8 inhibitor AMTB on the latency of seizures in PTZ‐induced acute seizure mice (*n*: Con = 15, AMTB = 12, 99.07 ± 9.24 s vs. 186.42 ± 27.66 s, *p* = 0.001). (D) Effect of intraperitoneal injection of the TRPM 8 inhibitor AMTB on the S2 latency of seizures in PTZ‐induced acute seizure mice (*n*: Con = 12, AMTB = 9, 139.73 ± 11.63 s vs. 267.67 ± 39.58 s, *p* = 0.003). (E) Effect of intraperitoneal injection of the TRPM 8 inhibitor AMTB on the S4 latency of seizures in PTZ‐induced acute seizure mice (*n*: Con = 11, AMTB = 9, 231.25 ± 35.79 s vs. 390.33 ± 55.39 s, *p* = 0.03). Bar graph represent mean ± SEM. *P < 0.05; **P < 0.01.

To verify the function of TRPM8 in seizures, AMTB, an inhibitor of TRPM8, was intraperitoneally injected in male mice, and the control group received the same amount of saline, and then the PTZ‐induced acute seizure model was developed in both groups and the differences in behavioral changes between them during seizures were assessed. Mice were observed and recorded for 30 min after injection of PTZ (60 mg/kg) to induce an acute seizure model (Figure 1A). The latency before seizure onset and the grade at seizure onset are important criteria that reflect the severity of seizures, which can clearly distinguish the behavioral differences between different groups; these two indicators were carefully observed and recorded. As a result, we found that inhibition of TRPM8 by AMTB alleviated the average seizure grade (*n*: Con = 15, AMTB = 12, 4.13 ± 0.22 vs. 3.42 ± 0.26, *p* = 0.03, Figure 1B). Meanwhile, the latency to generalized seizures was increased in the AMTB group compared to the control group (*n*: Con = 15, AMTB = 12, 99.07 ± 9.24 s vs. 186.42 ± 27.66 s, *p* = 0.001, Figure 1C). Moreover, the suppression of TRPM8 has a significant effect on the lengthening of S2 and S4 occurrence times during the progression of acute seizures compared to the control (*n*: Con = 12, AMTB = 9, 139.73 ± 11.63 s vs. 267.67 ± 39.58 s, *p* = 0.003, Figure 1D) (*n*: Con = 11, AMTB = 9, 231.25 ± 35.79 s vs. 390.33 ± 55.39 s, *p* = 0.03, Figure 1E). When TRPM8 is specifically inhibited, seizure latency is prolonged and seizure grade is reduced. The prolonged latency before seizure onset implies that inhibition of TRPM8 slows the threshold at which neuronal cells reach the abnormally firing potential, delaying the entire seizure progression; the decrease in seizure grade suggested that the duration and intensity of the convulsions and the damage to the body are reduced. To rule out the effect of sex on the experimental results, we repeated the experiment in female mice and observed that AMTB similarly reduced the mean grade of seizures and significantly shortened the latency period (Figure S2). These findings suggest that inhibiting TRPM8 may play a protective role in the development of seizures.

### 3.2 Knockdown of TRPM8 Consistently Exert an Ameliorative Effect on the Acute Seizure Progression

**FIGURE 2:**
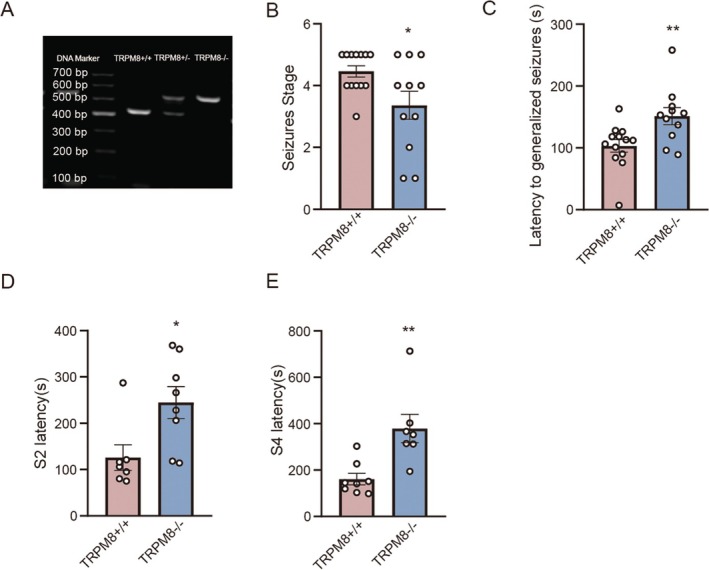
Knockdown of TRPM8 consistently exerts an ameliorative effect on the acute seizure progression. (A) Identification of genotypes of TRPM 8‐KO mice by PCR (WT:426 bp; TM:500 bp). (B) Changes of seizure stage of the TRPM8‐KO mice compared to wild type mice (*n*: TRPM8+/+ = 13, TRPM8−/− = 11, 4.46 ± 0.18 vs. 3.36 ± 0.45, *p* = 0.07). (C) Changes in seizure latency of TRPM8‐KO mice compared to wild type mice (*n*: TRPM8+/+ = 13, TRPM8−/− = 11, 103.23 ± 10.08 s vs. 151.36 ± 13.75 s, *p* = 0.009). (D) Changes in seizure S2 latency of TRPM8‐KO mice compared to wild type mice (*n*: TRPM8+/+ = 7, TRPM8−/− = 8, 125.71 ± 37.65 s vs. 244.75 ± 34.51 s, *p* = 0.02). (E) Changes in seizure latency of TRPM8‐KO mice compared to wild type mice (*n*: TRPM8+/+ = 8, TRPM8−/− = 7, 160.87 ± 70.07 s vs. 379.28 ± 161.20s, *p* = 0.004). Bar graph represent mean ± SEM. *P < 0.05; **P < 0.01.

To further confirm the role of TRPM8 in acute seizures, we constructed a PTZ‐induced acute seizure model in TRPM8 gene knockout (TRPM8−/−) male mice and evaluated the seizure stage and latency of the mice (Figure 2A). Compared to the TRPM8+/+ mice, the TRPM8−/− mice presented a lower seizure stage (*n*: TRPM8+/+ = 13, TRPM8−/− = 11, 4.46 ± 0.18 vs. 3.36 ± 0.45, *p* = 0.07, Figure 2B). Correspondingly, the latency to generalized seizures was increased in the TRPM8 −/− group (*n*: TRPM8+/+ = 13, TRPM8−/− = 11, 103.23 ± 10.08 s vs. 151.36 ± 13.75 s, *p* = 0.009, Figure 2C). The latency of S2 and S4 was also significantly increased in the TRPM8 −/− group compared to the control group (*n*: TRPM8+/+ = 7, TRPM8−/− = 8, 125.71 ± 37.65 s vs. 244.75 ± 34.51 s, *p* = 0.02, Figure 2D) (*n*: TRPM8+/+ = 8, TRPM8−/− = 7, 160.87 ± 70.07 s vs. 379.28 ± 161.20s, *p* = 0.004, Figure 2E). In experiments with female subjects, TRPM8 knockout mice were also observed to show a significant reduction in seizure grade and seizure latency compared with wild‐type mice (Figure S3). The development of seizures is negatively impacted by TRPM8 inhibition and complete knockout, which confirms that TRPM8 is involved in regulating seizures. These results proved that TRPM8 is crucial to the onset of acute seizure and participates in a certain part of the acute seizure progression.

### 3.3 The Expression of TRPM8, p‐CREB and p‐PKA Were Increased in the PFC of Mice With Seizures

In recent years, studies have shown that CREB is involved in the development of Parkinson's disease and Alzheimer's disease, suggesting that CREB has a central role in neurological disorders. It was found that inhibition of cAMP response element‐binding protein transcription in a seizure model shortened the duration of epileptic states and reduced the number of spontaneous seizures [29]. The TRPM8 core promoter region has been reported to contain a CRE that may be located between −1417 and −1406 base pairs. It may play an important role in promoting the transcription of TRPM8 [31, 32]. These studies suggest that TRPM8 may play a role in the regulation of seizures through the transcription factor CREB and its downstream signaling pathways.

**FIGURE 3:**
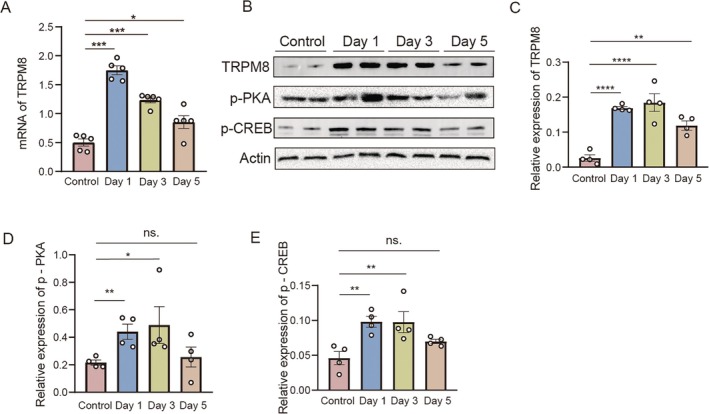
The expressions of TRPM8, p‐CREB, and p‐PKA were increased in the PFC of mice with seizures. (A) Statistics of TRPM8 mRNA expression in PFC of PTZ‐induced acute seizure mice (*n* = 5, Control = 0.50 ± 0.06, Day 1 = 1.75 ± 0.08, Day 3 = 1.24 ± 0.05, Day 5 = 0.85 ± 0.11, Control vs. Day1, *p* < 0.001, Control vs. Day3, *p* < 0.001, Control vs. Day5, *p* = 0.02). (B) Expression of TRPM8, p‐PKA and p‐CREB in the PFC of seizure mice, as assessed by western blotting. (C) Statistics of TRPM8 expression in PFC of PTZ‐induced acute seizure mice (*n* = 4, Control = 0.03 ± 0.01, Day 1 = 0.17 ± 0.004, Day 3 = 0.18 ± 0.03, Day 5 = 0.12 ± 0.01, Control vs. Day1, *p* < 0.0001, Control vs. Day3, *p* < 0.0001, Control vs. Day5, *p* = 0.003). (D) Statistics of p‐PKA expression in PFC of PTZ‐induced acute seizure mice (*n* = 4, Control = 0.22 ± 0.02, Day 1 = 0.44 ± 0.56, Day 3 = 0.49 ± 0.13, Day 5 = 0.26 ± 0.07, Control vs. Day1, *p* = 0.009; Control vs. Day3, *p* = 0.03; Control vs. Day 5, ns). (E) Statistics of p‐CREB expression in PFC of PTZ‐induced acute seizure mice (*n* = 4, Control = 0.05 ± 0.01, Day 1 = 0.10 ± 0.008, Day 3 = 0.10 ± 0.02, Day 5 = 0.07 ± 0.003, Control vs. Day1, *p* = 0.007, Control vs. Day3, *p* = 0.008, Control vs. Day 5, ns). Bar graph represent mean ± SEM. *P < 0.05; **P < 0.01; ***P < 0.001; ****P < 0.0001; ns: no significant difference.

Building on this mechanistic foundation, we investigated the temporal expression patterns of TRPM8 and its associated signaling molecules in a mouse model of epilepsy. We first measured TRPM8 mRNA levels in the prefrontal cortex (PFC) using qPCR and found a significant upregulation on day 1 and day 3 post‐seizure (*n* = 5, Control = 0.50 ± 0.06, Day 1 = 1.75 ± 0.08, Day 3 = 1.24 ± 0.05, Day 5 = 0.85 ± 0.11, Control vs. Day1, *p* < 0.001, Control vs. Day3, *p* < 0.001, Control vs. Day5, *p* = 0.02, Figure 3A). Furthermore, the expression of TRPM8, p‐PKA and p‐CREB were measured by western blotting in the PFC of normal and epileptic male mice during seizures. The results showed that the expression of TRPM8, p‐CREB and p‐PKA were significantly elevated on day 1 and 3 after seizures compared with the control group (Figure 3), which was also observed in female mice (Figure S4). Statistics of TRPM8, p‐PKA, p‐CREB expression in PFC of seizure mice (TRPM8: *n* = 4, Control = 0.03 ± 0.01, Day 1 = 0.17 ± 0.004, Day 3 = 0.18 ± 0.03, Day 5 = 0.12 ± 0.01, Control vs. Day1, *p* < 0.0001, Control vs. Day3, p < 0.0001, Control vs. Day5, *p* = 0.003.; p‐PKA: *n* = 4, Control = 0.22 ± 0.02, Day 1 = 0.44 ± 0.56, Day 3 = 0.49 ± 0.13, Day 5 = 0.26 ± 0.07, Control vs. Day1, *p* = 0.009, Control vs. Day3, *p* = 0.03, Control vs. Day5, ns.; p‐CREB: *n* = 4, Control = 0.05 ± 0.01, Day 1 = 0.10 ± 0.008, Day 3 = 0.10 ± 0.02, Day 5 = 0.07 ± 0.003, Control vs. Day1, *p* = 0.007, Control vs. Day3, *p* = 0.008, Control vs. Day5, ns.).

### 3.4 The Expression of TRPM8, p‐CREB and p‐PKA Were Increased in PTZ‐Induced Acute Seizure Cell Model

**FIGURE 4:**
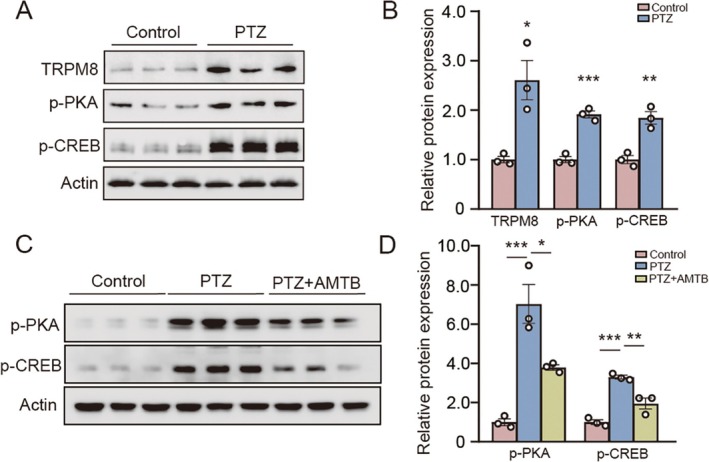
The expression of TRPM8, p‐CREB, and p‐PKA were increased in the PTZ‐induced acute seizure cell model. (A) Expression of TRPM8, p‐PKA, and p‐CREB in PTZ‐induced acute seizure cell model, as assessed by western blotting. (B) Statistics of TRPM8 expression in PTZ‐induced acute seizure cell model (*n* = 3, Control = 1.00 ± 0.06, PTZ = 2.61 ± 0.40, Control vs. PTZ, *p* = 0.016). Statistics of p‐PKA expression in PTZ‐induced acute seizure cell model (*n* = 3, Control = 1.00 ± 0.06, PTZ = 1.91 ± 0.07, Control vs. PTZ, *p* = 0.0006). Statistics of p‐CREB expression in PTZ‐induced acute seizure cell model (*n* = 3, Control = 1.00 ± 0.08, PTZ = 1.85 ± 0.13, Control vs. PTZ, *p* = 0.005). (C) Expression of p‐PKA and p‐CREB in acute seizure cell model treated with AMTB, as assessed by western blotting. (D) Statistics of p‐PKA expression in PTZ‐induced acute seizure cell model with AMTB (*n* = 3, Control = 1.00 ± 0.17, PTZ = 7.03 ± 0.99, PTZ + AMTB = 3.79 ± 0.14, Control vs. PTZ, *p* = 0.0007, PTZ vs. PTZ + AMTB, *p* = 0.016). Statistics of p‐CREB expression in PTZ‐induced acute seizure cell model with AMTB (*n* = 3, Control = 1.00 ± 0.11, PTZ = 3.30 ± 0.10, PTZ + AMTB = 1.95 ± 0.28, Control vs. PTZ, *p* = 0.0002, PTZ vs. PTZ + AMTB, *p* = 0.004).Bar graph represent mean ± SEM. *P < 0.05; **P < 0.01; ***P < 0.001.

To further validate our preliminary findings, we carefully reviewed the literature that established a PTZ‐induced acute seizure model in human neuroblastoma SH‐SY5Y cells, a well‐characterized neuronal model system for seizure research [33]. SH‐SY5Y cells were treated with 20 mM pentylenetetrazol (PTZ) for 48 h. Cellular experiments demonstrated that PTZ treatment significantly elevated the expression levels of TRPM8, phosphorylated PKA, and phosphorylated CREB (p‐CREB at Ser^133^) compared to control groups (Figure 4A,B; Statistics of TRPM8 expression: *n* = 3, Control = 1.00 ± 0.06, PTZ = 2.61 ± 0.40, Control vs. PTZ, *p* = 0.016; Statistics of p‐PKA expression: *n* = 3, Control = 1.00 ± 0.06, PTZ = 1.91 ± 0.07, Control vs. PTZ, *p* = 0.0006; Statistics of p‐CREB expression: *n* = 3, Control = 1.00 ± 0.08, PTZ = 1.85 ± 0.13, Control vs. PTZ, *p* = 0.005). It is worth noting that in the seizure cell model, the expressions of p‐PKA and p‐CREB significantly decreased after TRPM8 inhibitor AMTB treatment (Figure 4C,D; Statistics of p‐PKA expression in seizure cell model with AMTB: *n* = 3, Control = 1.00 ± 0.17, PTZ = 7.03 ± 0.99, PTZ+AMTB = 3.79 ± 0.14, Control vs. PTZ, *p* = 0.0007, PTZ vs. PTZ+AMTB, *p* = 0.016; Statistics of p‐CREB expression in seizure cell model with AMTB: *n* = 3, Control = 1.00 ± 0.11, PTZ = 3.30 ± 0.10, PTZ+AMTB = 1.95 ± 0.28, Control vs. PTZ, *p* = 0.0002, PTZ vs. PTZ+AMTB, *p* = 0.004). These findings provide further validation of our hypothesis that TRPM8 modulates seizures through the PKA/CREB pathway. As a calcium‐permeable channel, TRPM8 activation induces Ca^2+^influx, which directly promotes adenylate cyclase (AC) activation [12]. This leads to elevated intracellular cAMP levels, subsequently activating protein kinase A (PKA) [34, 35]. The catalytic subunit of PKA (PKAca) then dissociates from its regulatory subunits and translocates to the nucleus, where it phosphorylates CREB at the Ser^133^ residue—a key regulatory site essential for CREB transcriptional activity [23, 36]. Phosphorylated CREB (p‐CREB) ultimately modulates target gene transcription, thereby contributing to the pathogenesis of seizures.

### 3.5 TRPM8 Modulates Seizure via PKA/CREB Pathway

**FIGURE 5:**
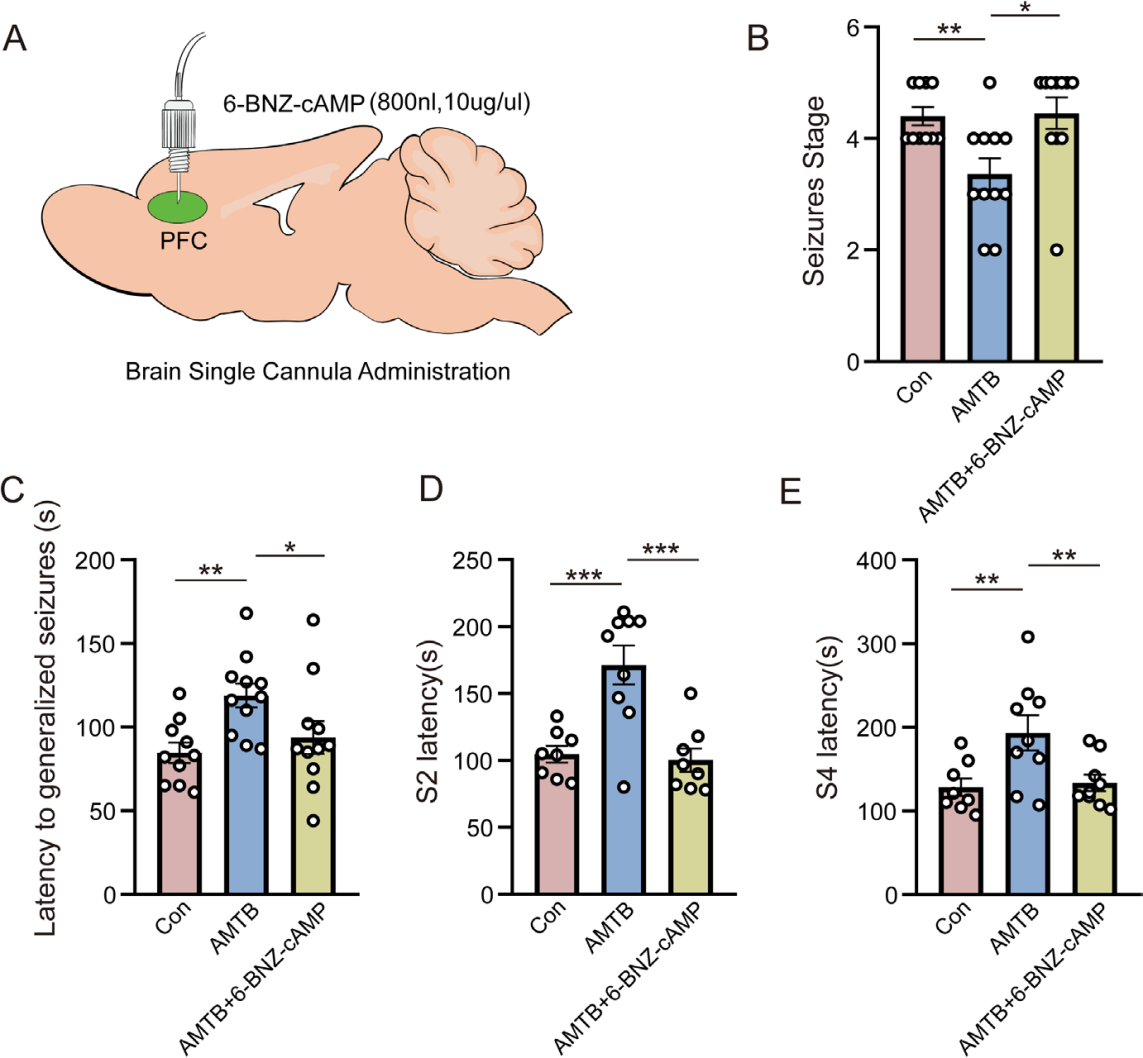
The activation of PKA in the brain manifested a reversed effect of inhibiting TRPM8. (A) Schematic representation of stereotaxic injection in the PFC brain region of mice. (B) Effect of PKA agonist injection on average seizure stage after inhibited TRPM8 (*n*: Con = 10, AMTB = 11, AMTB+6‐BNZ‐cAMP = 11, average seizure stage: Con = 4.4 ± 0.16, AMTB = 3.36 ± 0.28, AMTB+6‐BNZ‐cAMP = 4.46 ± 0.28, Con vs. AMTB, *p* = 0.005; AMTB vs. AMTB+6‐BNZ‐cAMP, *p* = 0.03). (C) Effect of PKA agonist injection on general seizure latency after TRPM8 inhibition in mice (*n*: Con = 10, AMTB = 11, AMTB+6‐BNZ‐cAMP = 11, Latency of generalized seizures: Con = 84.7 ± 6.04 s, AMTB = 118.91 ± 7.23 s, AMTB+6‐BNZ‐cAMP = 93.73 ± 9.83 s; Con vs. AMTB, *p* = 0.006; AMTB vs. AMTB+6‐BNZ‐cAMP, *p* = 0.01). (D) Effect of PKA agonist injection on epileptic S2 latency after TRPM8 inhibition in mice (*n*: Con = 8, AMTB = 9, AMTB+6‐BNZ‐cAMP = 8, S2 latency: Con = 104.75 ± 6.24 s, AMTB = 171.33 ± 14.63 s, AMTB+6‐BNZ‐cAMP = 100.25 ± 8.71 s, Con vs. AMTB, *p* = 0.0003; AMTB vs. AMTB+6‐BNZ‐cAMP, *p* = 0.0001). (E) Effect of PKA agonist injection on the S4 latency of seizures following TRPM8 inhibition in mice (*n*: Con = 11, AMTB = 9, AMTB+6‐BNZ‐cAMP = 9, S4 latency: Con = 128.38 ± 10.62 s, AMTB = 193.44 ± 21.19 s, AMTB+6‐BNZ‐cAMP = 133.56 ± 9.82 s, Con vs. AMTB, *p* = 0.006; AMTB vs. AMTB+6‐BNZ‐cAMP, *p* = 0.009). Bar graph represent mean ± SEM. *P < 0.05; **P < 0.01; ***P < 0.001.

To further validate the hypothesis that the TRPM8/PKA/CREB signaling pathway regulates seizures, we performed further behavioral validation. We utilized stereotactic injection of PKA agonist 6‐BNZ‐cAMP in PFC to activate expression of PKA during seizures (Figure 5A). The mice were divided into three groups: the first group was injected with saline as control, the second group was intraperitoneally injected with AMTB, and the third group was injected with 6‐BNZ‐cAMP into the PFC after intraperitoneal injection of AMTB. Thirty minutes later, the three groups of mice were subjected to PTZ to induce seizures simultaneously to evaluate the seizure status. As a result, mice treated with TRPM8 inhibitor AMTB showed longer pre‐seizure latency and lower seizure grade after seizure induction compared with control mice. However, the effect of AMTB was significantly reversed by 6‐BNZ‐cAMP, and the seizure latency (S2, S4) and average seizure stage were almost restored to the state before TRPM8 inhibition (*n*: Con = 10, AMTB = 11, AMTB+6‐BNZ‐cAMP = 11, average seizure stage: Con = 4.4 ± 0.16, AMTB = 3.36 ± 0.28, AMTB+6‐BNZ‐cAMP = 4.46 ± 0.28, Con vs. AMTB *p* = 0.005, AMTB vs. AMTB+6‐BNZ‐cAMP *p* = 0.03, Figure 5B) (*n*: Con = 10, AMTB = 11, AMTB+6‐BNZ‐cAMP = 11, Latency of generalized seizures: Con = 84.7 ± 6.04 s, AMTB = 118.91 ± 7.23 s, AMTB+6‐BNZ‐cAMP = 93.73 ± 9.83 s; Con vs. AMTB *p* = 0.006, AMTB vs. AMTB+6‐BNZ‐cAMP *p* = 0.01, Figure 5C) (*n*: Con = 8, AMTB = 9, AMTB+6‐BNZ‐cAMP = 8, S2 latency: Con = 104.75 ± 6.24 s, AMTB = 171.33 ± 14.63 s, AMTB+6‐BNZ‐cAMP = 100.25 ± 8.71 s, Con vs. AMTB *p* = 0.0003, AMTB vs. AMTB+6‐BNZ‐cAMP *p* = 0.0001, Figure 5D) (*n*: Con = 11, AMTB = 9, AMTB+6‐BNZ‐cAMP = 9, S4 latency: Con = 128.38 ± 10.62 s, AMTB = 193.44 ± 21.19 s, AMTB+6‐BNZ‐cAMP = 133.56 ± 9.82 s, Con vs. AMTB *p* = 0.006, AMTB vs. AMTB+6‐BNZ‐cAMP *p* = 0.009, Figure 5E). The activation of PKA in the PFC of female mice also showed a reversal effect on the inhibition of TRPM8, which was reflected in the return of seizure grade and latency to a level nearly consistent with that of normal mice (Figure S5). These results not only indicated the role of TRPM8/PKA/CREB pathway in the transmission of molecular information in the development of seizures, but also suggested that the activation of this pathway may aggravate the occurrence and development of seizures.

### 3.6 Knockdown of TRPM8 Attenuates Apoptosis of PFC Neurons in Mice With Acute Seizure Induced by PTZ

**FIGURE 6:**
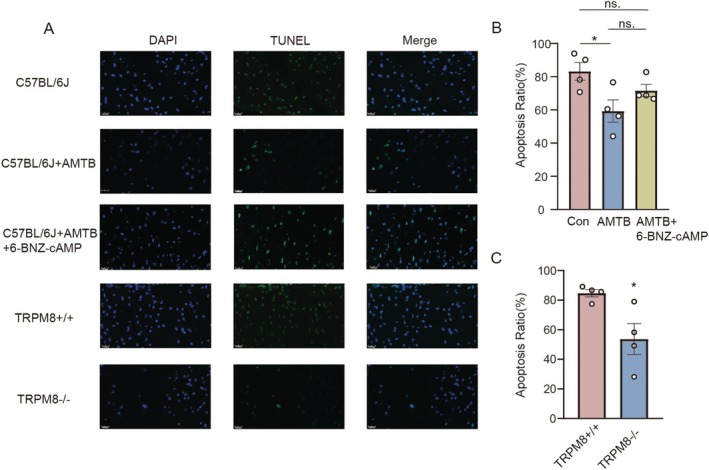
Inhibition of TRPM8 can alleviate neuronal apoptosis induced by seizures. (A) Representative images of TUNEL assay (original magnifications: ×40.0). (B) Changes in apoptotic cells with inhibited TRPM8 compared with control (*p* = 0.03). Changes in apoptotic cells after activation of PKA in mice after TRPM8 inhibition (ns). (C) Changes in apoptotic cells in TRPM8 knockout mice compared with wild type mice (*p* = 0.02). Bar graph represent mean ± SEM. *P < 0.05; ns: no significant difference.

Studies have shown that seizures can cause neuronal death, and in addition, seizures also activate neuronal apoptotic pathways. Based on the above results, we hypothesized that deficiency of TRPM8 could rescue the apoptosis of neuronal cells of PFC in seizure mice. TUNEL staining was used to evaluate the effect of TRPM8 on cell apoptosis during seizures. The mice were divided into five groups: C57+PTZ; C57+AMTB+PTZ; C57+AMTB+PTZ+6‐BANZ‐cAMP; TRPM8WT+PTZ; TRPM8KO+PTZ and were respectively observed the apoptotic cells in the PFC region of the brain. The results showed that inhibition or knockdown of TRPM8 indeed attenuated the level of apoptosis in the PFC of seizure mice. Either inhibition or knockdown of TRPM8 significantly reduced the proportion of TUNEL‐positive cells in the PFC of seizure mice. Mice with suppressed TRPM8, after activation of the prefrontal PKA, showed no significant difference in apoptotic cells compared to normal mice after PTZ‐induced acute seizure (Figure 6). Our findings indicated that low activity or low levels of TRPM8 have a negative effect on PFC neuronal apoptosis, suggesting that TRPM8 may be involved in the entire process of seizures by promoting neuronal apoptosis, including pre‐seizure latency, seizure intensity, and duration.

## 4 Discussion

The main findings of this study are as follows: First, inhibition or knockdown of TRPM8 delayed seizures, suggesting that TRPM8 is involved in seizures. Second, the levels of TRPM8, p‐PKA, and p‐CREB were significantly elevated in the prefrontal cortex during the seizure period or PTZ‐induced acute seizure cell model. Moreover, activation of PKA reversed the inhibitory effect of TRPM8 in seizures, suggesting that TRPM8 regulates seizures through the CREB/PKA pathway. Furthermore, TUNEL staining showed that downregulation or inhibition of TRPM8 reversed neuronal damage caused by seizures. Taken together, our work confirms the involvement of TRPM8 in the acute process of seizures through the regulation of PKA/CREB signaling pathway: As a calcium channel, activation of TRPM8 induces Ca^2+^ influx, which directly promotes adenylate cyclase (AC) activation; this leads to elevated intracellular cAMP levels [12, 13]. Subsequently, cAMP activates protein kinase A (PKA), triggering the dissociation of the PKA catalytic subunit from its regulatory subunits and its translocation to the nucleus [34]. PKA activation is solely mediated by cAMP‐induced allosteric regulation, releasing catalytically competent subunits, whereas constitutive phosphorylation of Thr^197^ in the subunit is essential for its structural integrity. The activated PKA catalytic subunit phosphorylates CREB at the Ser^133^ residue—a key regulatory site essential for CREB transcriptional activity [23, 36]. Phosphorylated CREB (p‐CREB) then modulates gene transcription, thereby participating in the pathogenesis of seizures.

TRPM8 was known for its essential role in the detection of cold in humans that has been found to participate in many diseases [37]. It is distributed not only in specific organs, but also in the peripheral nervous system and some regions of the brain, such as the hippocampus, amygdala, and prefrontal lobe [14]. Therefore, its function in the nervous system was worthy and needed to be explored. Interestingly, some recent studies paid attention to the role of TRPM8 in seizures. Moriyama H reported that activation of TRPM8 by the agonist Icilin performed a suppressive effect on seizures [38]. However, another study from Zandi N showed that inhibition M8‐B of TRPM8 rescued the seizures induced by PTZ [20]. Accordingly, whether TRPM8 has a positive or negative effect on seizures was still ambiguous. Therefore, our study applied the TRPM8‐specific antagonist AMTB and TRPM8 knockout mice to explore the role of TRPM8 in acute seizure models, respectively. This result further confirmed the positive effect of inhibiting or knocking down TRPM8 on seizure control. Meanwhile, we applied AMTB to the PTZ‐induced acute seizure model for the first time, which is important for the development of antiepileptic drugs.

The PFC was a crucial brain area for controlling learning, memory, emotion, social behavior, and other mental processes. It had the ability to dynamically integrate inputs from other brain areas to encode and control advanced cognitive processes [39]. Multiple atrophy of the cerebral cortex in the prefrontal lobe was observed in patients with epileptic syndrome, which was associated with impairment of overall cognition, memory, and processing speed [40]. Concerning the fact that TRPM8 was detected to express in the PFC, we exerted to unveil the potential characteristic of TRPM8 of PFC in seizures. To understand the role of TRPM8 in seizures, we used western blot assay and found that the expression level of TRPM8 was up‐regulated in the PFC of PTZ‐induced mice on day 1 and 3, which suggested that TRPM8 has an important role in seizure processes. Studies have concluded that the TRPM8 activation accompanied with Ca^2+^ influx and protein kinase A (PKA) stimulation in adipocyte [41]. As the downstream molecule of PKA, CREB had widely functioned in the nervous system such as synaptic plasticity, spatial memory, long term memory [24]. Previous studies had recognized the significance of CREB. For the development of seizures, over expression of CREB had an excitatory effect, whereas inhibition or deficiency of CRBE presents an inhibitory effect. Meanwhile, increased CREB level was detected up to the following 7 days of seizures [29, 30]. Moreover, CREB phosphorylation peaked between 3 and 8 min following the start of seizure activity and then gradually declines [42]. A current study on acute myeloid leukemia (AML) highlighted a relationship between TRPM8 and CREB, which indicated a regulative role of TRPM8 on CREB. In view of this, we intended to find out if the same action of TRPM8 has a modulating influence on seizures. Thus, we also performed the Western blotting to assess the level of p‐PKA and p‐CREB. The findings demonstrated that p‐CREB considerably increased on day 1 and 3 following seizures. This finding further explained TRPM8's role in seizures. Except for the evidence in the molecular levels, ulteriorly behavioral validation has coincided with the change of protein levels. The stereotactic injection of PKA agonist 6‐BNZ‐cAMP in PFC reversed the effect of AMTB in mice undergoing the PTZ‐induced acute seizure model, which firmly supported that TRPM8 modulates seizure via PKA/CREB pathway.

Seizures were confirmed by experimental researches and clinical evidence that accompanied brain cell loss, especially for prolonged seizures [43, 44]. The death of neuronal cells has been linked to the occurrence of apoptosis, a programmed process of cell death that contributes to the stabilization of homeostasis. Necroptosis, pyroptosis, ferroptosis, and autophagy are also shown to be the main approaches for cell death [45, 46]. Moreover, during the pathological conditions that are deleterious to the nervous system, the impact of neuronal cell loss in the hippocampus is the most profound [44]. Aiming to find out whether TRPM8's inhibition has an effect on the loss of neuronal cells, tunnel staining was conducted in the PTZ‐induced acute seizure model. Our works presented that more severe cell death occurred in the TRPM8‐WT group than the TRPM8‐KO group, which is accordant with previous findings that the PTZ‐induced acute seizure model does damage to the neuronal cells [47, 48]. If mice with TRPM8 inhibition are activated by the PKA in the prefrontal cortex, the proportion of apoptotic cells will increase to a certain extent again after the acute seizure. Studies on the neuronal damage of seizures could provide evidence for the exploration of protective drugs for the deleterious effect on the brain. Combined with the previous experimental results, we proposed that TRPM8 may be involved in the seizure process of mice by regulating neuronal apoptosis through the PKA/CREB signaling pathway.

Notable sexual dimorphism characterizes both the epidemiology and pathophysiology of epilepsy. These disparities arise from inherent neurobiological differences in brain morphology, structure, and functional connectivity between sexes and may also stem from alterations in endogenous hormone levels across various life stages [49, 50]. Significantly, antiepileptic interventions demonstrate bidirectional interactions with hormonal cycles, while endogenous/exogenous hormones reciprocally modulate seizure susceptibility in epileptic patients. In this investigation, we systematically examined the sexual conservation of TRPM8‐mediated seizure regulation using a rigorously controlled experimental paradigm. Female subjects underwent standardized protocols under precisely matched conditions to eliminate confounding sex‐related variables, thereby enhancing the translational validity of therapeutic evaluation. Our expanded analysis revealed remarkable conservation of TRPM8‐dependent seizure regulation across both sexes. Findings demonstrate that: (1) TRPM8 inhibition/knockout in female mice significantly attenuated seizure severity (*p* < 0.01) and prolonged seizure latency (*p* < 0.05). (2) Prefrontal cortex activation of TRPM8‐downstream signaling molecules in TRPM8‐inhibited females restored seizure activity to near‐baseline levels. This conserved mechanism across sexes provides critical insights for developing universal seizure therapeutics while emphasizing the necessity of sex‐controlled experimental designs in neuropharmacological research. The preserved regulatory capacity of TRPM8 signaling despite hormonal variations suggests its fundamental role in seizure pathophysiology beyond endocrine‐mediated effects.

Although our research has revealed a new mechanism by which the TRPM8/PKA/CREB signaling pathway regulates seizures, we have not yet obtained data on the expression or function of TRPM8 from clinical samples or public databases, which requires further research in the future. PTZ‐induced acute seizure model's value lies in probing acute seizure mechanisms rather than epilepsy disease‐modification. PTZ‐induced acute seizures primarily model generalized tonic–clonic seizures but fail to replicate the neuropathological hallmarks of chronic seizure (e.g., mossy fiber sprouting, neuronal loss, or gliosis), limiting translational relevance to human epileptogenesis [51]. This model effectively screens acute antiseizure drugs (ASDs) targeting ion channels/neurotransmitter receptors. However, it exhibits low predictive value for anti‐epileptogenic therapies that modify disease progression, as spontaneous recurrent seizures and comorbidities are absent [52]. Future studies should prioritize utilizing chronic seizure models to screen for compounds with antiepileptogenic, disease‐modifying, or neuroprotective effects, thereby addressing the limitations of acute seizure models.

In summary, our results confirm the role of TRPM8 in the PTZ‐induced acute seizure model, reveal the possible mechanism by which TRPM8 regulates seizures in mice through the PKA/CREB pathway, and provide a potential target for seizure treatment; but, of course, differences between different seizure models must be taken into account, and the roles of TRPM8 in different models need to be further explored.

## Author Contributions

Jia‐Zhan Huang: validation, data curation, investigation, resources, visualization, software. Gui‐Feng Lu: conceptualization, methodology, formal analysis, investigation, data curation, writing – original draft, funding acquisition. Yi‐Han Jiang and Yao Guo: Validation, data curation, investigation. Ze‐Yu Lin: investigation. Zhi Zhang: software. Fei Geng: writing – reviewing and editing, visualization, supervision, project administration, funding acquisition.

## Funding

This work was supported by the National Natural Science Foundation of China (82300064), Guangdong Basic and Applied Basic Research Foundation (2023A1515010874 and 2024A1515011099), SUMC Scientific Research Initiation Grant (510858057 and 510858067), SUMC College Students’ Innovative Entrepreneurial Training Plan Program (202310560043).

## Ethics Statement

All animal procedures performed in this study were conducted in strict compliance with the international standards of Animal Care Guidelines and have been approved by the Institutional Animal Ethics Committee of Shantou University Medical College (SUMCSY2024‐005).

## Conflicts of Interest

The authors declare no conflicts of interest.

## Supporting information


**Figure S1:** Flow diagram of the mouse behavioral experiment.

**Figure S2:** Inhibition of TRPM8 mitigated the progression of acute seizures in female mice. (A) Effect of intraperitoneal injection of the TRPM8 inhibitor AMTB on acute seizures stage in PTZ‐induced acute seizure mice (*n*: Con = 15, AMTB = 17, 4.73 ± 0.11 vs. 3.82 ± 0.21, *p* = 0.003). (B) Effect of intraperitoneal injection of the TRPM 8 inhibitor AMTB on the latency of seizures in PTZ‐induced acute mice (*n*: Con = 15, AMTB = 17, 74.13 ± 3.61 s vs. 141.00 ± 5.52 s, *p* = 0.0001). (C) Effect of intraperitoneal injection of the TRPM 8 inhibitor AMTB on the S2 latency of seizures in PTZ‐induced acute mice (*n*: Con = 15, AMTB = 17, 103.40 ± 5.571 s vs. 207.40 ± 9.70s, *p* = 0.0001). (D) Effect of intraperitoneal injection of the TRPM 8 inhibitor AMTB on the S4 latency of seizures in PTZ‐induced acute mice (*n*: Con = 15, AMTB = 16, 201.90 ± 16.68 s vs. 283.90 ± 19.05 s, *p* = 0.03).

**Figure S3:** Knockdown of TRPM8 consistently exert a ameliorative effect on the acute seizure progression in female mice. (A) Changes of seizure stage of the TRPM8 KO mice compared to wild type mice (*n*: TRPM8+/+ = 15, TRPM8−/− = 15, 4.67 ± 0.13 vs. 4.07 ± 0.18, *p* = 0.02). (B) Changes in seizure latency of TRPM 8 KO mice compared to wild type mice (*n*: TRPM8+/+ = 15, TRPM8−/− = 15, 89.33 ± 5.28 s vs. 123.3 ± 7.19 s, *p* = 0.0007). (C) Changes in seizure S2 latency of TRPM 8 KO mice compared to wild type mice (*n*: TRPM8+/+ = 15, TRPM8−/− = 15, 197.90 ± 6.35 s vs. 281.1 ± 8.13 s, *p* = 0.0001). (D) Changes in seizure latency of TRPM 8 KO mice compared to wild type mice (*n*: TRPM8+/+ = 15, TRPM8−/− = 15, 214.70 ± 14.27 s vs. 287.10 ± 10.19 s, *p* = 0.0007).

**Figure S4:** The expressions of TRPM8, p‐CREB and p‐PKA were increased in the PFC of mice with seizures. (A) Expression of TRPM8, p‐PKA and p‐CREB in the PFC of seizure mice, as assessed by western blotting. (B) Statistics of TRPM8 expression in PFC of seizure mice (*n* = 4, Control = 0.35 ± 0.02, Day1 = 0.56 ± 0.06, Day3 = 0.48 ± 0.03, Day5 = 0.31 ± 0.05, Control vs. Day1, *p* = 0.028; Control vs. Day3, *p* = 0.01; Control vs. Day5, ns.). (C) Statistics of p‐PKA expression in PFC of seizure mice (*n* = 4, Control = 0.23 ± 0.02, Day 1 = 0.42 ± 0.03, Day3 = 0.33 ± 0.06, Day5 = 0.27 ± 0.03, Control vs. Day1, *p* = 0.028; Control vs. Day3, *p* = 0.34; Control vs. Day5, ns.). (D) Statistics of p‐CREB expression in PFC of seizure mice (*n* = 4; Control = 0.32 ± 0.05, Day1 = 0.79 ± 0.14, Day3 = 0.81 ± 0.14, Day5 = 0.68 ± 0.17, Control vs. Day1, *p* = 0.28; Control vs. Day3, *p* = 0.026; Control vs. Day5, ns).

**Figure S5:** The activation of PKA in brain manifested a reversed effect of inhibition TRPM8 in female mice. (A) Effect of PKA agonist injection on average seizure stage after inhibited TRPM8 (*n*: Con = 10, AMTB = 10, AMTB+6‐BNZ‐cAMP = 10, average seizure stage: Con = 4.90 ± 0.10, AMTB = 3.80 ± 0.25, AMTB+6‐BNZ‐cAMP = 4.60 ± 0.22, Con vs. AMTB, *p* = 0.001; AMTB vs. AMTB+6‐BNZ‐cAMP, *p* = 0.017). (B) Effect of PKA agonist injection on general seizure latency after TRPM8 inhibition in mice (*n*: Con = 10, AMTB = 10, AMTB+6‐BNZ‐cAMP = 10, Latency of generalized seizures: Con = 91.80 ± 5.79 s, AMTB = 111.40 ± 4.98 s, AMTB + 6‐BNZ‐cAMP = 92.70 ± 4.79 s; Con vs. AMTB, *p* = 0.02; AMTB vs. AMTB+6‐BNZ‐cAMP, *p* = 0.03). (C) Effect of PKA agonist injection on seizure S2 latency after TRPM8 inhibition in mice (*n*: Con = 10, AMTB = 10, AMTB+6‐BNZ‐cAMP = 10, S2 latency: Con = 129.30 ± 3.87 s, AMTB = 188.20 ± 9.86 s, AMTB+6‐BNZ‐cAMP = 126.10 ± 5.65 s, Con vs. AMTB, *p* = 0.001; AMTB vs. AMTB+6‐BNZ‐cAMP, *p* = 0.0002). (D) Effect of PKA agonist injection on the S4 latency of seizures following TRPM8 inhibition in mice (*n*: Con = 10, AMTB = 10, AMTB+6‐BNZ‐cAMP = 10, S4 latency: Con = 229.20 ± 9.72 s, AMTB = 281.40 ± 9.10 s, AMTB+6‐BNZ‐cAMP = 217.9 ± 13.14 s, Con vs. AMTB, *p* = 0.004; AMTB vs. AMTB+6‐BNZ‐cAMP, *p* = 0.0006).

**Data S1:** cns70709‐sup‐0006‐DataS1.doc.

## Data Availability

All data within the study are presented in the paper and/or the Supporting Information. The original data and additional data related to this study are available from the corresponding author upon reasonable request.
