# Supplementary figures and images for: Prefrontal TRPM8 Receptor Modulates Epileptic Seizures via PKA/CREB Signaling Pathway in Mice
Source: CNS Neurosci Ther. 2025 Dec 31;32(1):e70709. doi: 10.1002/cns.70709 (PMC12755055; doi:10.1002/cns.70709)

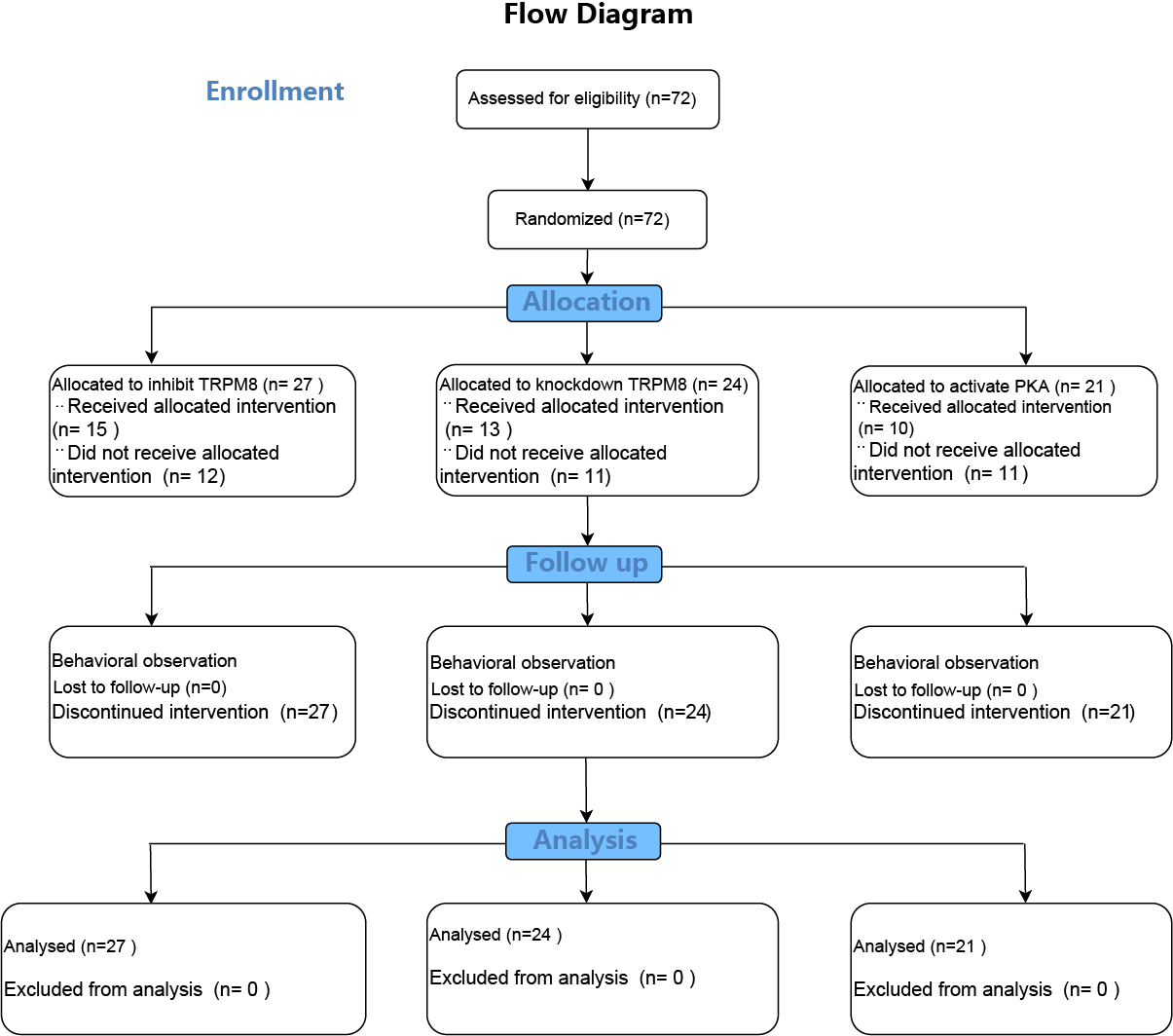

Supplement: Supplementary file 1 — Figure S1: Flow diagram of the mouse behavioral experiment. [file CNS-32-e70709-s005.tif]

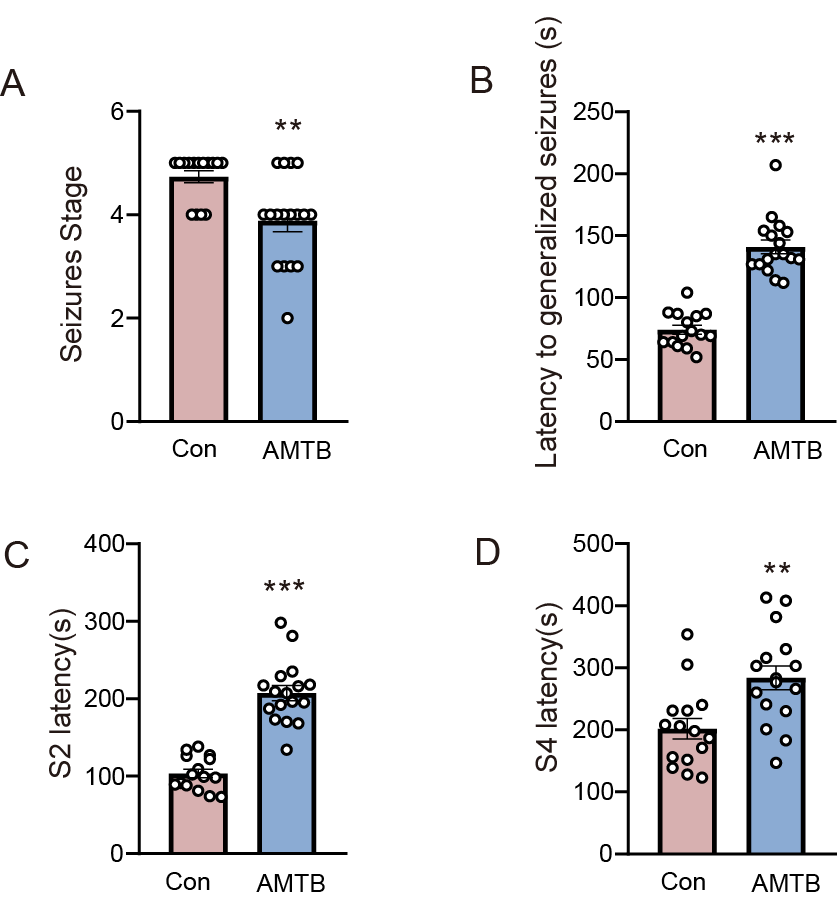

Supplement: Supplementary file 2 — Figure S2: Inhibition of TRPM8 mitigated the progression of acute seizures in female mice. (A) Effect of intraperitoneal injection of the TRPM8 inhibitor AMTB on acute seizures stage in PTZ‐induced acute seizure mice (n: Con = 15, AMTB = 17, 4.73 ± 0.11 vs. 3.82 ± 0.21, p = 0.003). (B) Effect of intraperitoneal injection of the TRPM 8 inhibitor AMTB on the latency of seizures in PTZ‐induced acute mice (n: Con = 15, AMTB = 17, 74.13 ± 3.61 s vs. 141.00 ± 5.52 s, p = 0.0001). (C) Effect of intraperitoneal injection of the TRPM 8 inhibitor AMTB on the S2 latency of seizures in PTZ‐induced acute mice (n: Con = 15, AMTB = 17, 103.40 ± 5.571 s vs. 207.40 ± 9.70s, p = 0.0001). (D) Effect of intraperitoneal injection of the TRPM 8 inhibitor AMTB on the S4 latency of seizures in PTZ‐induced acute mice (n: Con = 15, AMTB = 16, 201.90 ± 16.68 s vs. 283.90 ± 19.05 s, p = 0.03). [file CNS-32-e70709-s006.tif]

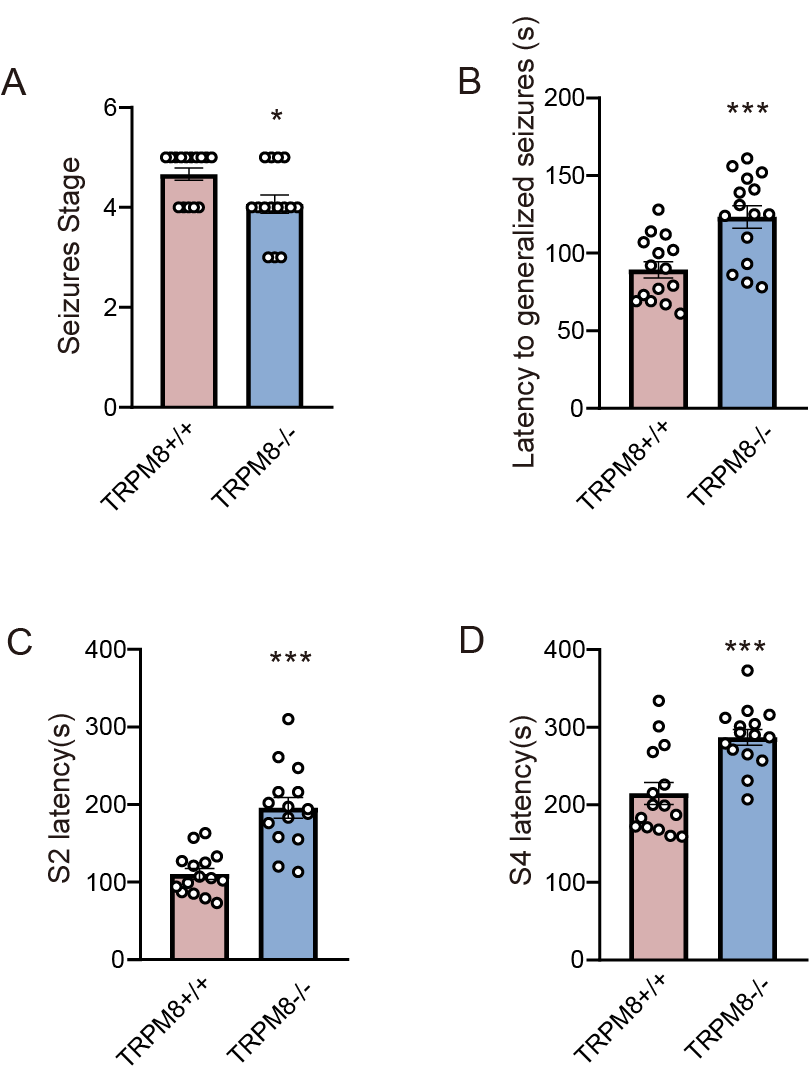

Supplement: Supplementary file 3 — Figure S3: Knockdown of TRPM8 consistently exert a ameliorative effect on the acute seizure progression in female mice. (A) Changes of seizure stage of the TRPM8 KO mice compared to wild type mice (n: TRPM8+/+ = 15, TRPM8−/− = 15, 4.67 ± 0.13 vs. 4.07 ± 0.18, p = 0.02). (B) Changes in seizure latency of TRPM 8 KO mice compared to wild type mice (n: TRPM8+/+ = 15, TRPM8−/− = 15, 89.33 ± 5.28 s vs. 123.3 ± 7.19 s, p = 0.0007). (C) Changes in seizure S2 latency of TRPM 8 KO mice compared to wild type mice (n: TRPM8+/+ = 15, TRPM8−/− = 15, 197.90 ± 6.35 s vs. 281.1 ± 8.13 s, p = 0.0001). (D) Changes in seizure latency of TRPM 8 KO mice compared to wild type mice (n: TRPM8+/+ = 15, TRPM8−/− = 15, 214.70 ± 14.27 s vs. 287.10 ± 10.19 s, p = 0.0007). [file CNS-32-e70709-s001.tif]

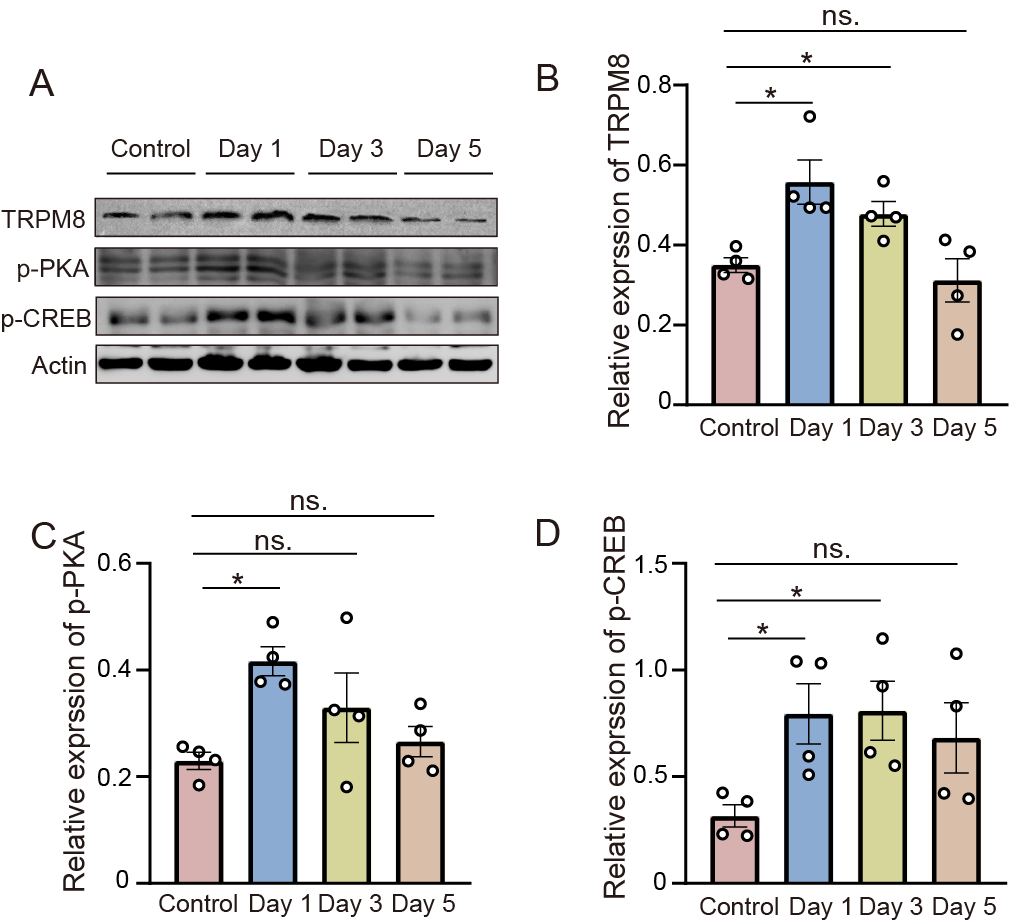

Supplement: Supplementary file 4 — Figure S4: The expressions of TRPM8, p‐CREB and p‐PKA were increased in the PFC of mice with seizures. (A) Expression of TRPM8, p‐PKA and p‐CREB in the PFC of seizure mice, as assessed by western blotting. (B) Statistics of TRPM8 expression in PFC of seizure mice (n = 4, Control = 0.35 ± 0.02, Day1 = 0.56 ± 0.06, Day3 = 0.48 ± 0.03, Day5 = 0.31 ± 0.05, Control vs. Day1, p = 0.028; Control vs. Day3, p = 0.01; Control vs. Day5, ns.). (C) Statistics of p‐PKA expression in PFC of seizure mice (n = 4, Control = 0.23 ± 0.02, Day 1 = 0.42 ± 0.03, Day3 = 0.33 ± 0.06, Day5 = 0.27 ± 0.03, Control vs. Day1, p = 0.028; Control vs. Day3, p = 0.34; Control vs. Day5, ns.). (D) Statistics of p‐CREB expression in PFC of seizure mice (n = 4; Control = 0.32 ± 0.05, Day1 = 0.79 ± 0.14, Day3 = 0.81 ± 0.14, Day5 = 0.68 ± 0.17, Control vs. Day1, p = 0.28; Control vs. Day3, p = 0.026; Control vs. Day5, ns). [file CNS-32-e70709-s004.tif]

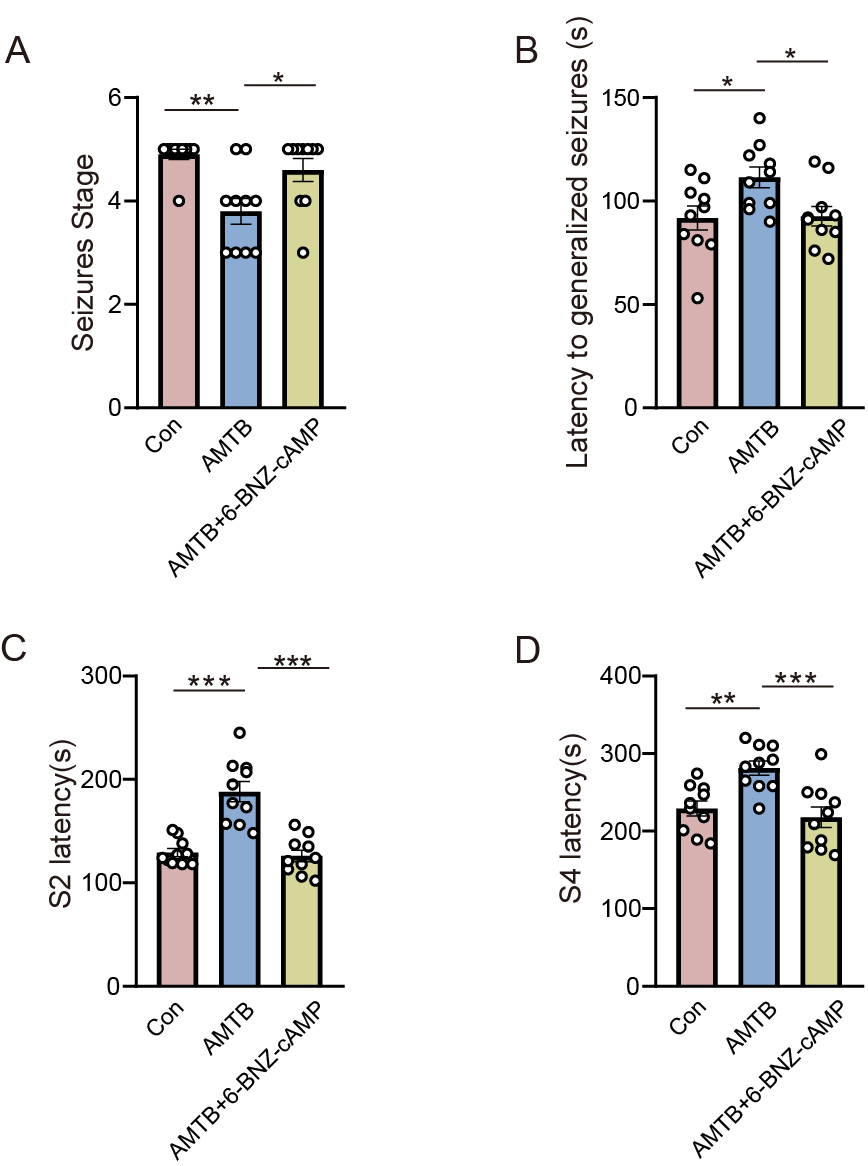

Supplement: Supplementary file 5 — Figure S5: The activation of PKA in brain manifested a reversed effect of inhibition TRPM8 in female mice. (A) Effect of PKA agonist injection on average seizure stage after inhibited TRPM8 (n: Con = 10, AMTB = 10, AMTB+6‐BNZ‐cAMP = 10, average seizure stage: Con = 4.90 ± 0.10, AMTB = 3.80 ± 0.25, AMTB+6‐BNZ‐cAMP = 4.60 ± 0.22, Con vs. AMTB, p = 0.001; AMTB vs. AMTB+6‐BNZ‐cAMP, p = 0.017). (B) Effect of PKA agonist injection on general seizure latency after TRPM8 inhibition in mice (n: Con = 10, AMTB = 10, AMTB+6‐BNZ‐cAMP = 10, Latency of generalized seizures: Con = 91.80 ± 5.79 s, AMTB = 111.40 ± 4.98 s, AMTB + 6‐BNZ‐cAMP = 92.70 ± 4.79 s; Con vs. AMTB, p = 0.02; AMTB vs. AMTB+6‐BNZ‐cAMP, p = 0.03). (C) Effect of PKA agonist injection on seizure S2 latency after TRPM8 inhibition in mice (n: Con = 10, AMTB = 10, AMTB+6‐BNZ‐cAMP = 10, S2 latency: Con = 129.30 ± 3.87 s, AMTB = 188.20 ± 9.86 s, AMTB+6‐BNZ‐cAMP = 126.10 ± 5.65 s, Con vs. AMTB, p = 0.001; AMTB vs. AMTB+6‐BNZ‐cAMP, p = 0.0002). (D) Effect of PKA agonist injection on the S4 latency of seizures following TRPM8 inhibition in mice (n: Con = 10, AMTB = 10, AMTB+6‐BNZ‐cAMP = 10, S4 latency: Con = 229.20 ± 9.72 s, AMTB = 281.40 ± 9.10 s, AMTB+6‐BNZ‐cAMP = 217.9 ± 13.14 s, Con vs. AMTB, p = 0.004; AMTB vs. AMTB+6‐BNZ‐cAMP, p = 0.0006). [file CNS-32-e70709-s003.tif]
